# Supplementary material for: Biodentine Stimulates Calcium-Dependent Osteogenic Differentiation of Mesenchymal Stromal Cells from Periapical Lesions
Source: Int J Mol Sci. 2025 Apr 29;26(9):4220. doi: 10.3390/ijms26094220 (PMC12072047; doi:10.3390/ijms26094220)
Supplement: Supplementary file 1 [file ijms-26-04220-s001.zip › ijms-3552045-supplementary figure.pdf]

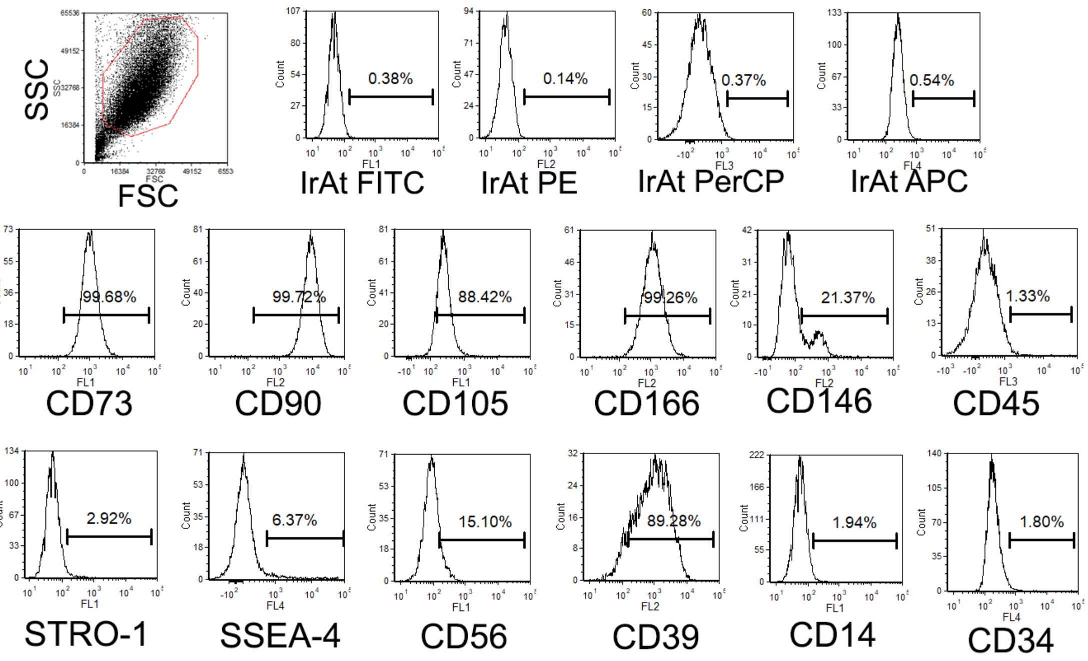

Supplementary Figure S1. Histogram display of fluorescence for one PL-MSC line. The percentage of positive cells after labeling with specific monoclonal antibodies conjugated with fluorochromes is shown within the designated FSC/SSC profile of the analyzed cells. Gates indicate the level of specific fluorescence and were set based on controls with the corresponding irrelevant monoclonal antibodies (IrAb).
