# Supplementary material for: Biodentine Stimulates Calcium-Dependent Osteogenic Differentiation of Mesenchymal Stromal Cells from Periapical Lesions
Source: Int J Mol Sci. 2025 Apr 29;26(9):4220. doi: 10.3390/ijms26094220 (PMC12072047; doi:10.3390/ijms26094220)
Supplement: Supplementary file 1 [file ijms-26-04220-s001.zip › ijms-3552045-supplementary table.pdf]

Supplementary Table S1. Essential Functions of Genes Involved in Osteoblast Differentiation and Bone Metabolism.

**Gene**	**Essential Functions in Osteoblast Differentiation and Bone Metabolism**
RUNX2	Master transcription factor that drives osteoblast differentiation, activating osteogenic genes like COL1A1 and SP7; essential for bone matrix production and mineralization.
SP7 (Osterix)	Key regulator of osteoblast maturation, controlling the expression of genes involved in bone matrix production, such as COL1A1 and ALP.
WNT2	Activates the Wnt/β-catenin pathway, promoting osteoblast lineage commitment, proliferation, and bone formation.
COL1A1	Encodes type I collagen, the main component of the bone extracellular matrix, providing structural integrity and scaffolding for mineralization.
BMP-2	Induces osteoblast differentiation through SMAD signaling, enhancing bone matrix deposition and mineralization.
BGLAP (Osteocalcin)	Marker of late-stage osteoblast differentiation; involved in bone mineralization and regulation of metabolic processes.
TGF-β1	Modulates osteoblast differentiation, proliferation, and bone remodeling through extracellular matrix regulation.
FGF-2	Stimulates osteoblast proliferation, differentiation, and bone regeneration.
ALP (Alkaline Phosphatase)	Hydrolyzes phosphate esters, increasing phosphate availability for hydroxyapatite formation and bone mineralization.
FOSB	Regulates osteoblast differentiation and response to mechanical stress through the AP-1 transcription complex.
FOSL2	Modulates osteoblast activity, bone remodeling, and interactions between osteoblasts and osteoclasts.
DDR1	Involved in collagen signaling, affecting osteoblast adhesion, migration, and extracellular matrix remodeling.

Gene	Essential Functions in Osteoblast Differentiation and Bone Metabolism
DDR2	Regulates osteoblast proliferation, collagen deposition, and bone integrity.
CTGF (CCN2)	Enhances osteoblast differentiation, extracellular matrix production, and bone healing.
VCAN (Versican)	Modulates extracellular matrix organization, osteoblast adhesion, and bone matrix assembly.
RANKL	Regulates osteoclastogenesis and bone remodeling by stimulating osteoclast differentiation.
CTSK (Cathepsin K)	Plays a role in bone matrix degradation, osteoblast-osteoclast communication, and bone remodeling.
IL-6ST (gp130)	Mediates IL-6 signaling, influencing osteoblast differentiation, bone metabolism, and inflammatory responses.
PTHR (Parathyroid Hormone Receptor)	Mediates PTH signaling, stimulating osteoblast activity, bone formation, and calcium homeostasis.
GHR (Growth Hormone Receptor)	Regulates osteoblast proliferation and differentiation, promoting bone growth and remodeling.
